# CT findings as predictive factors for treatment failure in *Mycobacterium abscessus* complex lung disease: a retrospective cohort study

**DOI:** 10.1007/s11604-024-01570-y

**Published:** 2024-05-06

**Authors:** Pin-Yi Chiang, Yu-Sen Huang, Yu-Cheng Huang, Ming-Yann Lee, Victor Jing-Wei Kang, Chin-Chung Shu, Yeun-Chung Chang

**Affiliations:** 1 Department of Medical Imaging, National Taiwan University Hospital, Taipei, Taiwan; 2 Department of Medical Imaging, National Taiwan University Hospital Yun-Lin Branch, Yunlin, Taiwan; 3 Department of Internal Medicine, National Taiwan University Hospital, No 7, Chung Shan South Road, Taipei, Taiwan

**Keywords:** Nontuberculous mycobacteria, *Mycobacterium abscessus* complex lung disease, Computed tomography, Thorax, Treatment outcome

## Abstract

**Purpose:**

*Mycobacterium abscessus* complex (MABC) commonly causes lung disease (LD) and has a high treatment failure rate of around 50%. In this study, our objective is to investigate specific CT patterns for predicting treatment prognosis and monitoring treatment response, thus providing valuable insights for clinical physicians in the management of MABC-LD treatment.

**Methods:**

We retrospectively assessed 34 patients with MABC-LD treated between January 2015 and December 2020. CT scores for bronchiectasis, cellular bronchiolitis, consolidation, cavities, and nodules were measured at initiation and after treatment. The ability of the CT scores to predict treatment outcomes was analyzed in logistic regression analyses.

**Results:**

The CT scoring system had excellent inter-reader agreement (all intraclass correlation coefficients, > 0.82). The treatment failure (TF) group (17/34; 50%) had higher cavitation diameter (*p* = 0.049) and extension (*p* = 0.041) at initial CT and higher cavitation diameter (*p* = 0.049) and extension (*p* =0 .045), consolidation (*p* = 0.022), and total (*p* = 0.013) scores at follow-up CT than the treatment success (TS) group. The changes of total score and consolidation score (*p* = 0.049 and 0.024, respectively) increased in the TF group more than the TS group between the initial and follow-up CT. Multivariable logistic regression analysis showed initial cavitation extension, follow-up consolidation extension, and change in consolidation extension (adjusted odds ratio: 2.512, 2.495, and 9.094, respectively, per 1-point increase; all *p* < 0.05) were significant predictors of treatment failure.

**Conclusions:**

A high pre-treatment cavitation extension score and an increase in the consolidation extension score during treatment on CT could be alarm signs of treatment failure requiring tailor the treatment of MABC-LD carefully.

**Supplementary Information:**

The online version contains supplementary material available at 10.1007/s11604-024-01570-y.

## Introduction

Non-tuberculous mycobacteria (NTM) are previously recognized as non-pathogenic environmental bacteria, and the clinical significance of their associated diseases was neglected until the mid-twentieth century. Methodological advances in the mycobacteriology laboratory have contributed to an increasing awareness of NTM diseases during the last two decades. Over 200 species have been identified to date, many of which are reported to cause human diseases in pulmonary and extrapulmonary sites [1]. NTM lung disease (NTM-LD) is the most common manifestation of NTM infections [2]. Compared to tuberculosis (TB), NTM-LD is less virulent and not as contagious, but is more difficult to treat and exhibits multiple drug resistance [1]. As a result, NTM infections are difficult to effectively control and their global incidence has increased year-by-year in recent decades [2–4].

*Mycobacterium abscessus* complex (MABC) was first isolated in 1952 and further divided into three subspecies: *M. abscessus subsp. abscessus*, *M. abscessus subsp. bolletii*, and *M. abscessus subsp. massiliense.* MABC ranks as the second most common cause of NTM-LD globally, following *Mycobacterium avium* complex (MAC), although there are regional variations [5]. MABC is a rapidly growing mycobacteria with intrinsic and extensive resistance to antibiotics [6, 7], and has been regarded as a chronic incurable disease. Notably, treatment of MABC-LD is extremely challenging with a high failure rate of around 50–60% [8]. In addition, the efficacy of many drugs is not reliable, except for clarithromycin and amikacin. Therefore, it may be useful to modify the treatment regimens for individual patients with MABC-LD according to treatment response by radiographical monitor [1].

In fact, initial radiographic findings have been reportedly associated with treatment outcomes in NTM-LD [9]. For MABC-LD, common radiographic findings include cellular bronchiolitis, bronchiectasis, nodules, cavities, and consolidation [7, 10–14]. However, despite the lengthy course (> 1 year) and high failure rate of treatment, there are few indicators and scarce reports regarding radiographic findings that could be used to tailor the treatment of MABC-LD during the treatment [14, 15]. In a previous report [14], patients with MABC-LD had poorer CT responses compared to patients with *M. massiliense* lung disease, but details of the findings that indicate treatment failure in MABC-LD were not studied. Therefore, in this study, we aimed to quantify and analyze the serial changes in CT findings for a range of lung abnormalities in patients with MABC-LD before and after/during treatment and investigate the ability of these characteristics to predict the treatment response.

## Materials and methods

### Design, setting and patients

This retrospective cohort study was conducted at a tertiary referral medical center, and one of its local branch in Taiwan. This study was approved by the institutional review boards (IRB No. 202106154RIND). The requirement for informed consent was waived due to the retrospective design. Adult (≥ 20 years) patients who met the American Thoracic Society/Infectious Diseases Society of America diagnostic criteria for MABC-LD and received standard treatment (at least two effective drugs) between January 2015 and December 2020 were enrolled [1]. The exclusion criteria were (1) computed tomography (CT) scans before or after/during treatment not available, (2) superimposed *Mycobacterium tuberculosis* infection, (3) mixed infection with other NTM, (4) previous organ transplant, (5) active cancer, receiving chemotherapy or immunotherapy, or (6) autoimmune disease with long-term use of steroids or disease modifying anti-rheumatic drugs.

### Definitions, outcome and CT scans

We recorded patient’s age, gender, body mass index (BMI), underlying disease, respiratory symptoms, and sputum acid-fast smear and mycobacterial culture results. Sputum mycobacterial culture were suggested to examine every 1–3 months if sputum mycobacterial culture had not yet conversion. Anti-MABC treatment was prescribed according to treatment guideline and adjusted by patient’s tolerance and clinical judgement [1]. Microbiological cure was defined as negative conversion in sputum at the end of treatment [16]. We classified patients into the treatment success (TS) group and treatment failure (TF) group based on whether microbiological cure was achieved or not.

CT scans were obtained using a helical technique; CT scanners including four 64-detector row (SOMATOM Sensation64 and SOMATOM definition AS, SIEMENS; Discovery CT 750 HD and Revolution EVO, GE Healthcare), a 128-detector row (Ingenuity CT, Philips Healthcare), two 256-detector row (iCT, Philips Healthcare; Revolution CT, GE Healthcare) and two 320-detector row (Aquilion ONE ViSION, Canon) were used with a pitch range 0.9–1.3. The CT acquisition parameters were as follows: 120–140 kVp; 100–200 mA. The reconstruction interval was 5 mm and the section thicken was 5 mm for axial images and 3 mm for coronal and sagittal images using soft kennel. High-resolution computed tomography (HRCT) was reconstructed for every patient in 1 mm thicknesses at 10-mm intervals using a sharp kennel. Contrast medium was not administered to every patient. Patients were imaged from the levels of the lung apices to the lung bases in the supine position and inspired during the scan.

### CT interpretation and semi-quantitative analysis of lesions

Every patient had ≥ two CT scan during their treatment period; we only analyzed two CT scans for each patient in this study. The initial CT was the scan closest to the start of treatment, and the follow-up CT scan was obtained 18 months after treatment started with some exceptions. These exceptions are due to early termination of treatment, thus no more CT was performed after 18 months. The CT scans were evaluated independently by two thoracic radiologists, one with 14 years and the other with 7 years of CT interpretation experience. The radiologists were blinded to each patient’s clinical information and treatment outcome. The whole cases were scored completely within 6 months.

Six lobes were evaluated in each patient (the lingula division of the left upper lobe was regarded as a separate lobe) and assessed for the presence of parenchymal abnormalities, including bronchiectasis, cellular bronchiolitis, cavities, nodules (10‒30 mm in diameter), and consolidation.

Bronchiectasis was defined as abnormal dilation of the bronchial lumen larger than the adjacent pulmonary arteries without tapering. Cellular bronchiolitis was defined in imaging features of centrilobular nodules (< 10 mm) and branching structures [17]. The presence of other abnormalities, including cavities, nodules (10–30 mm in diameter) and consolidation, was also recorded.

The CT scores reflecting the severity of lung involvement were calculated using a modified version of the scoring system published by Kim et al. [14]. Scores were given by considering the presence, severity, and extension of bronchiectasis, cellular bronchiolitis, cavities, nodules, and consolidation. A score of 0–3 was assigned to each item and a total score of 0–24 was summed up. The left upper lobe was divided into the left upper lobe and left lingula lobe. The severity of bronchiectasis is measured by the ratio of bronchus diameter to adjacent vessel diameter, while the severity of bronchiolitis is assessed by the peripheral to central distribution. The extension of all patterns was estimated by the involved proportion of each lobe’s segments. Further details are elaborated in Table 1.

**Table 1:** The scoring system of computed tomography (CT) for the assessment of the extent of *Mycobacterium abscessus* complex lung disease

CT findings	Score 0	Score1	Score 2	Score 3
Bronchiectasis (6 points)				
Severity^a^	Absent	Mild	Moderate	Severe
Lesion extension^c^	Absent	1–5	6–9	> 9
Cellular bronchiolitis (6 points)				
Severity^b^	Absent	Mild	Moderate	Severe
Lesion extension^c^	Absent	1–5	6–9	> 9
Cavitation (6 points)				
Diameter (cm)d	Absent	< 3	3–5	> 5
Lesion extension^c^	Absent	1–2	3–4	> 4
Consolidation (3 points)^c^	Absent	1–3	4–5	> 5
Nodules (3 points)^c^	Absent	1–2	3–4	> 4

^a^Mild = bronchus diameter is around 1.5–2 times that of adjacent vessel; moderate = bronchus diameter 2–3 times vessel diameter; severe = bronchus diameter > three times vessel diameter

^b^Mild = identifiable, peripheral lung, 1 cm from pleura; moderate = definite, involvement greater than 1–3 cm from pleura; severe = extensive, extending to central lung

^c^Involvement of each of the six lobes was estimated as follows: 0, no involvement; 1, involving less than 33.3% of the total number of segments in the lobe; 2, involving 33.4–66.6% of the total number of segments in the lobe; 3, involving greater than 66.7% of the total number of segments in the lobe. Total score ranges from 0 to 18 (each lobe has a maximum score of three and there are total of six lobes)

^d^The largest cavitation is evaluated, and the largest outer diameter (cm) is recorded as follows: 0 for no cavitation, 1 for outer diameter < 3 cm, 2 for outer diameter ≥ 3 cm and ≤ 5 cm, and 3 for outer diameter > 5 cm

After evaluating the severity and distribution of various patterns, the disease CT patterns were classified into three categories: fibrocavitary, nodular bronchiectatic, and unclassifiable forms. The fibrocavitary form is characterized by upper lobe cavitary disease, fibrosis and pleural thickening. The nodular bronchiectatic form exhibits bronchiectasis and cellular bronchiolitis predominantly in the middle lobe and lingular segments. Cases that do not fit into either the fibrocavitary or nodular bronchiectatic forms are classified as unclassifiable.

Both qualified and experienced thoracic radiologists interpreted all CT scans using the scoring system. They were blinded to the clinical data and each other’s interpretations.

### Statistical analyses

IBM SPSS statistical package (version 26.0, IBM Corp., Armonk, NY, USA) was used for statistical analyses. The comparisons between the TS and TF group were performed using the Fisher exact test for categorical variables and Mann–Whitney *U* test for continuous variables. The Wilcoxon pair test was used to compare the scores before and after treatment. Logistic regression was used to analyze factors associated with treatment outcome. Multivariable analysis included the factors that were statistically significant in univariate analysis. Receiver operating characteristic (ROC) curves were plotted to examine the predictive ability of the independent factors identified in logistic regression analysis. The Youden index was applied to set cutoff values.

## Results

### Characteristics of the patients

**Fig. 1:**
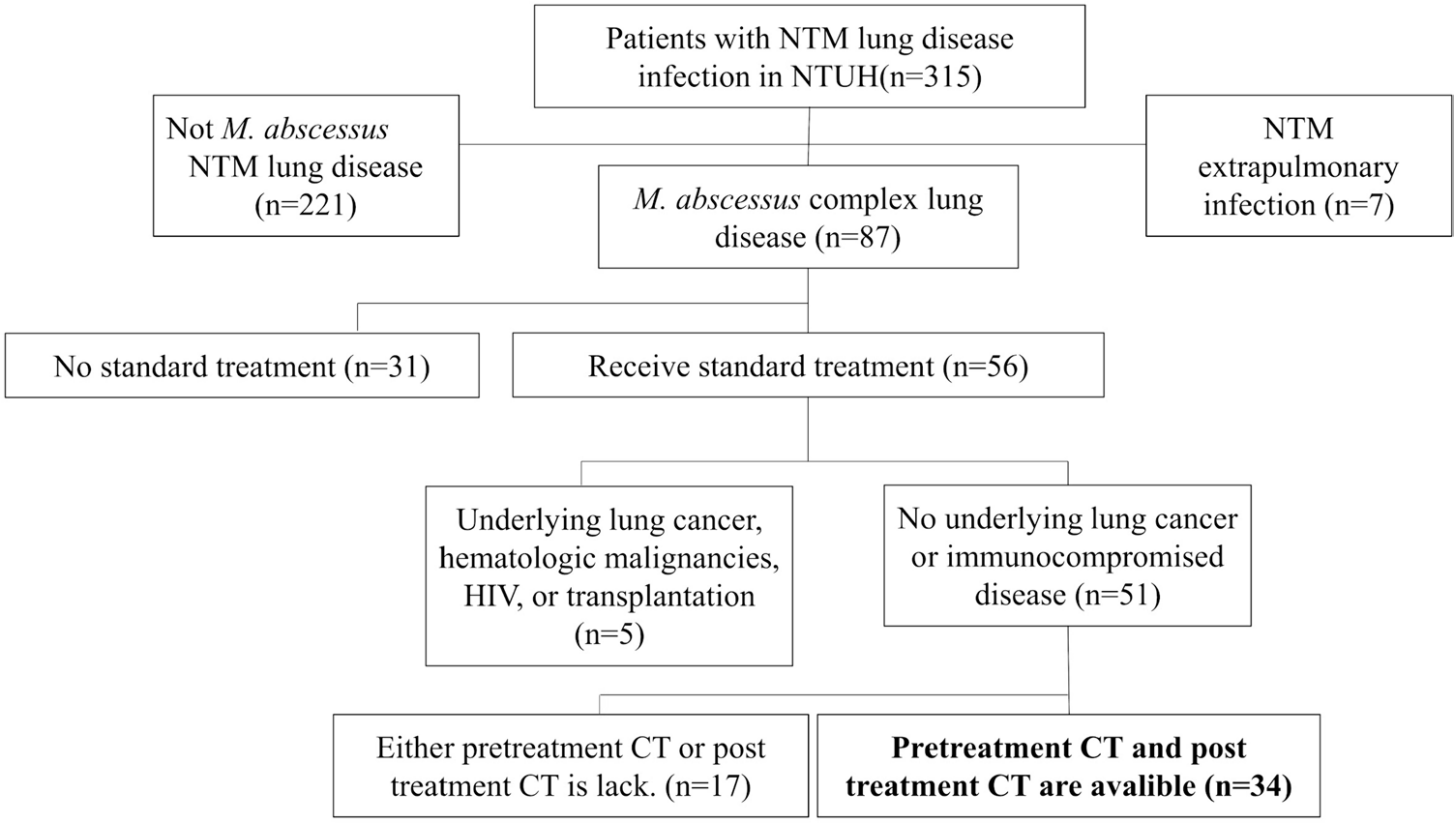
Flowchart of patient enrolment. Abbreviations: *M. abscessus*, *Mycobacterium abscessus* complex; NTM, nontuberculous mycobacteria; HIV, human immunodeficiency virus; CT, computed tomography; NTUH, National Taiwan University Hospital

We retrospectively reviewed the pre- and post-treatment CT scans for a total of 34 patients with MABC-LD who received anti-MABC treatment (Fig. 1). The median age was 58 years [median (IQR): 58 (52–70)] and females accounted for 88% (*n* = 30) of patients (Table 2). There was no significant between-group difference regarding age, sex, BMI, respiratory symptoms, sputum AFS titer for MABC, subspecies of MABC, underlying disease and treatment regimen (Table 2). The *Mycobacterium massiliense* accounted for 12.5% (*n* = 4) of the patients and *Mycobacterium abscessus* for the remaining 87.5% (*n* = 28). There was no difference between the TF and TS groups although the strains from two of the participants were lost. The median treatment time was 13 months and was insignificantly longer in the TF group compared with the TS group (26 vs. 12 months, *p* = 0.17). Sputum conversion was not achieved in 17 of the 34 patients (50%) who were classified into the treatment failure (TF) group. The remaining 17 patients who achieved negative sputum conversion were categorized into the treatment success (TS) group.

**Table 2:** Baseline characteristics of patients with *Mycobacterium abscessus* complex lung disease according to treatment outcome

Characteristics	All patients (*n* = *34*)	TS group (*n* = *17*)	TF group (*n* = *17*)	*p* valve
Age, year	58 (52–70)	54 (48–69)	62 (54–72)	0.274
Sex, female	30 (88%)	14 (82%)	16 (94%)	0.287
Body mass index, kg/m^2^	19.59 (18.22–21.45)	20.16 (18.67–22.72)	19.58 (17.73–20.6)	0.259
Respiratory symptoms				
Cough	26 (76.5%)	12 (71%)	14 (82%)	0.688
Hemoptysis	9 (26.5%)	5 (29%)	4 (24%)	1
SOB	8 (23.5%)	3 (17.6%)	5 (29%)	0.688
Fever	6 (17.6%)	3 (17.6%)	3 (18%)	1
Positive AFS smear	28 (82.4%)	13 (76.5%)	15 (88%)	0.312
MABC subspecies^a^, no.	32	16	16	1
*Mycobacterium abscessus*	28 (87.5%)	14 (87.5%)	14 (87.5%)	
*Mycobacterium massiliense*	4 (12.5%)	2 (12.5%)	2 (12.5%)	
Type of disease				
Fibro-cavitary	6 (17.6%)	1 (5.9%)	5 (29.4%)	0.135
Nodular bronchiectasis	27 (79.4%)	15 (88.2%)	12 (70.6%)	
Unclassifiable form	1 (2.9%)	1 (5.9%)	0 (0%)	
Underlying disease				
Diabetes	2 (6%)	2 (12%)	0	0.145
Active cancer	5 (15%)	3 (18%)	2 (12%)	0.832
COPD	1 (3%)	0	1 (6%)	0.310
Prior TB	5 (15%)	1 (6%)	4 (24%)	0.146
Autoimmune disease	4 (12%)	3 (18%)	1 (6%)	0.287
Treatment				
IV medication	8 (24%)	5 (30%)	3 (18%)	0.419
Macrolide containing	31 (91%)	15 (88%)	16 (94%)	0.287
Treatment duration, months	13 (6–30.75)	12 (6–18.5)	26 (5.5–44.5)	0.131

Data are median (IQR), or NO (%) or other indicated

^a^The total number for MABC subspecies data was 32, but the two of them could not be obtained due to retrospective limitations

Definition of abbreviations: AFB = Acid-fast bacilli; COPD = chronic obstructive pulmonary disease; IQR = interquartile range; IV = intravenous; SOB = shortness of breath; TB = tuberculosis; TS = treatment success, sputum conversion was achieved; TF = treatment failure, sputum conversion was not achieved; MABC = *Mycobacterium abscessus* complex

Overall, 27 (79.4%) of patients had the nodular bronchiectasis form of MABC-LD, six (17.6%) had the fibrocavitary form, and the remaining one (2.9%) had an unclassifiable form. The TF group contained a higher proportion of cases with the fibrocavitary form than the TS group (five and one, respectively). However, the overall distribution of the three forms of MABC-LD was not significantly different between the two groups (*p* = 0.13).

### Initial CT pattern frequency and score

The inter-rater correlations for the CT scores indicated excellent inter-rater agreement (all Pearson correlations > 0.8; Table S1 in the supplementary file). The results are analyzed based on the interpretations from the two radiologists (Chiang PY and Huang YS).

The initial CT scans were obtained in a median [IQR] of 1.5 [0–6.25] months before the beginning of treatment, with no difference between the two groups [3 (0–12) months for the TF group vs. 1 (0–3.5) months for the TS group; *p* = 0.17].

The pattern of parenchymal findings and their frequencies on initial CT scans were described in Table 3. The most common initial CT findings were cellular bronchiolitis (*n* = 34, 100%), followed by consolidation (*n* = 31, 94.1%), bronchiectasis (*n* = 31, 91.2%), nodules (*n* = 28, 82.35%), and cavitation (*n* = 12, 35.3%). There was a statistically significant difference between the TF and TS groups in cavitation (*p* = 0.031, *n* = 9 in the TF group vs. *n* = 3 in the TS group).

**Table 3:** The pattern of the parenchymal findings and frequency at initial computed tomography (CT) according to treatment outcome

CT pattern	Total (*n* = 34)	TS group (*n* = 17)	TF group (*n* = 17)	*p* valve
Bronchiectasis	31 (91.2%)	15	16	0.545
Cellular bronchiolitis	34 (100%)	17	17	1
Cavitary	12 (35.3%)	3	9	**0.031**
Consolidation	32 (94.1%)	16	16	1
Nodules	28 (82.35%)	15	13	0.368

**Fig. 2:**
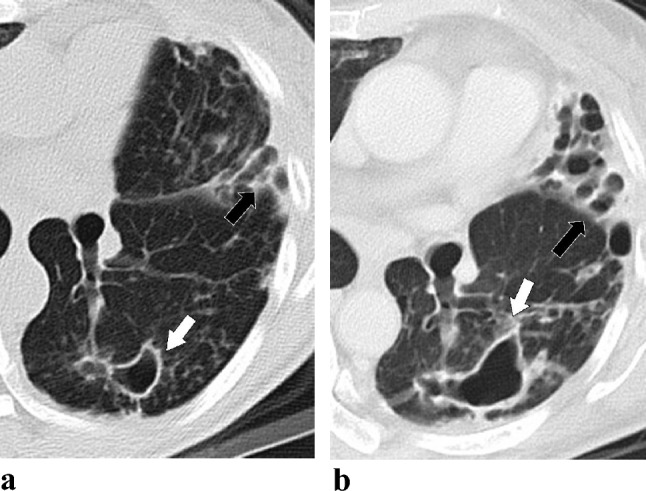
Serial changes in cavitating lung lesions in a 53-year-old female with *Mycobacterium abscessus* complex lung disease (MABC-LD) in the treatment failure group (without microbiological conversion). The follow-up period was 2 years. **a** Initial CT scans (section thickness, 5 mm, in axial plane) revealed a thin wall cavitation (white arrows) in the left lower lobe with severe bronchiectasis (black arrows) in the lingula left upper lobe. The largest diameter of cavitation is 5.44 cm in the apical left upper lobe (not shown), scoring 3 in cavitation diameter. For cavitation extension, involvement of two segments of the left upper lobe and one segment of the lingula lobe (not shown) earned 2 and 2 points, respectively. Additionally, involvement of all segments of the left lower lobe earned 3 points. This totals 7 points. Therefore, the final cavitation extension score is 3. **b** Follow-up CT scan (section thickness, 5 mm, in axial plane) at a similar level showed enlargement of the cavity and the bronchiectasis

The CT scores were evaluated using the modified system (Table 1) and listed the results in Table 4. Among the parameters, the initial median cavitation diameter (Fig. 2) and extension scores were significantly higher in the TF group than TS group. [1 (0–1) vs. 0 (0–0) for cavitation diameter, *p* = 0.049; 1 (0–2) vs. 0 (0–0) for cavitation extension, *p* = 0.041]. No other significant between-group differences were observed in the initial CT scores.

**Table 4:** The scores of the initial and the follow-up computed tomography (CT) according to treatment outcome in *Mycobacterium abscessus* complex lung disease

CT parameters /scores	TS group (*n* = 17)	TF group (*n* = 17)	*p* valve
Initial CT			
Bronchiectasis (6 points)	2 (2–5)	5 (2.5–5)	0.076
Cellular bronchiolitis (6 points)	5 (3.5–6)	2 (3–5.5)	0.08
Cavitation diameter (3 points)	0 (0–0)	1 (0–1)	**0.049**
Cavitation extension (3 points)	0 (0–0)	1 (0–2)	**0.041**
Consolidation (3 points)	1 (1–2.5)	2 (1–2)	0.454
Nodular (3 points)	2 (1–2)	1 (0.5–2)	0.205
Total Score (24 points)	12 (8–14)	13 (10–16)	0.136
Follow-up CT at least 18 months			
Bronchiectasis (6 points)	2 (2–5)	5 (3–5)	0.080
Cellular bronchiolitis (6 points)	5 (3–6)	5 (4–5)	0.831
Cavitation diameter (3 points)	0 (0–0.5)	1 (0–2)	**0.049**
Cavitation extension (3 points)	0 (0–0.5)	1 (0–2.5)	**0.045**
Consolidation (3 points)	1 (1–2)	2 (1.5–3)	**0.022**
Nodular (3 points)	1 (0–2)	1 (0.5–2)	0.843
Total score (24 points)	11 (8–12.5)	14 (10.5–19)	**0.013**
Changes between two scans^a^			
Bronchiectasis (6 points)	0 (− 1–1)	1 (0–3)	0.496
Cellular bronchiolitis (6 points)	0 (− 1–0)	0 (0–5)	0.205
Cavitation diameter (3 points)	0 (0–0)	0.33 (0–1.05)	0.059
Cavitation extension (3 points)	0 (0–0)	0 (0–0)	0.633
Consolidation (3 points)	0 (− 5–0)	0 (0–1)	**0.024**
Nodular (3 points)	0 (− 1–0)	0 (− 5–1)	0.131
Total score (24 points)	0 (− 1–1)	1 (0.5–2.5)	**0.049**

Values are presented as median (IQR). Boldface indicates *p* values less than 0.05

Comparison between the two groups using Mann–Whitney *U* test

CT, computed tomography; TS, treatment success; TF, treatment failed

^a^The score difference is score in follow-up CT minuses that in initial CT

### Follow-up CT scores and changes between the two scans

The follow-up CT scans were obtained in a median [IQR] of 22.5 [14.75–31.5] months after the start of treatment, with no difference between groups [27 (18.5–34) months for the TF group vs. 19 (10–32) months for the TS group; *p* = 0.17]. The median total score in the follow-up CT was significantly higher in TF group than TS group (*p* = 0.013). In addition, the TF group had significantly higher median scores for cavitation diameter, cavitation extension and consolidation (*p* = 0.049, *p* = 0.045 and *p* = 0.022, respectively).

**Fig. 3:**
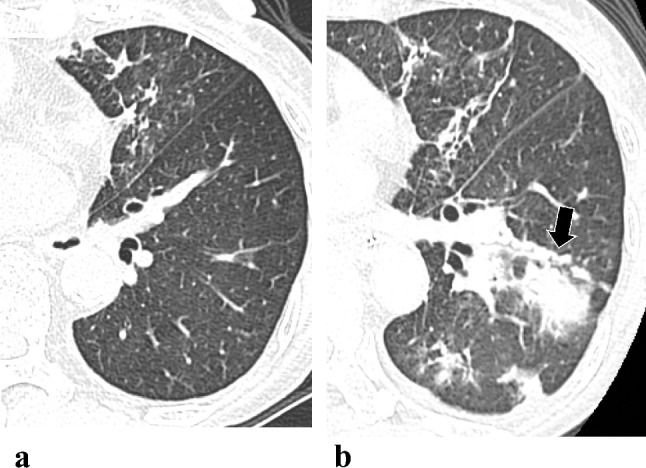
Serial changes in consolidative lung lesions in an 80-year-old female with *Mycobacterium abscessus* complex lung disease (MABC-LD) in the treatment failure group (without microbiological conversion). **a** Initial CT (section thickness, 5 mm, axial plane) showed no lung abnormalities in the left lower lobe. For the consolidation extension scoring in this patient, involvement of all segments of the entire right lung (not shown) earned 9 points, with no involvement in the left lung. This totals 9 points. Therefore, the final consolidation extension score is 3. **b** The follow-up scan revealed new multifocal, patchy consolidation in the left lower lobe. For the consolidation extension scoring in this image, involvement of one segment of the right upper lobe and all segments of the right middle and lower lobes (not shown) earned 7 points. Involvement of one subsegment of apicoposterior segment of the left upper lobe (LUL) earned 1 point, and involvement of one subsegment of inferior segment of the lingula LUL (not shown) earned 1 point. Additionally, involvement of all segments of the left lower lobes earned 3 points. This totals 12 points. Therefore, the final consolidation extension score is 3

**Fig. 4:**
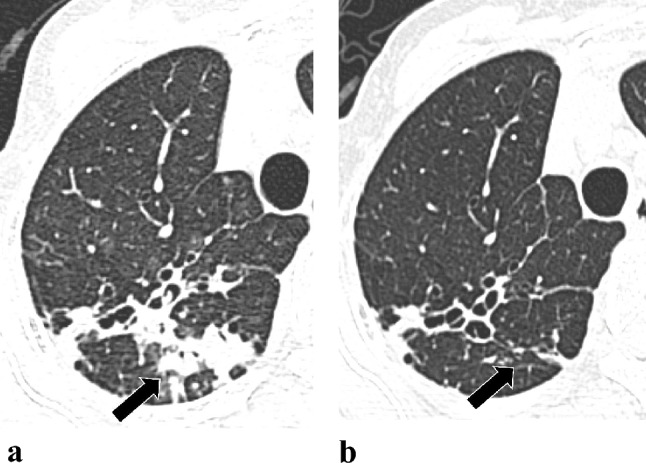
Serial changes in consolidative lung lesions in a 63-year-old female with *Mycobacterium abscessus* complex lung disease (MABC-LD) in the treatment success group (with microbiological conversion). **a** Initial CT (section thickness, 5 mm, in axial plane) revealed multifocal patchy consolidation in the right upper lobe. For the consolidation extension scoring in this patient, involvement of one segment of RUL earned 1 point; involvement of all segments of RML (not shown) earned 3 points; and involvement of 1/2 segment of the lingula LUL (not shown) earned 1 point. This totals 5 points. Therefore, the final consolidation extension score is 2. **b** The consolidation had mostly resolved at the follow-up scan after treatment

**Fig. 5:**
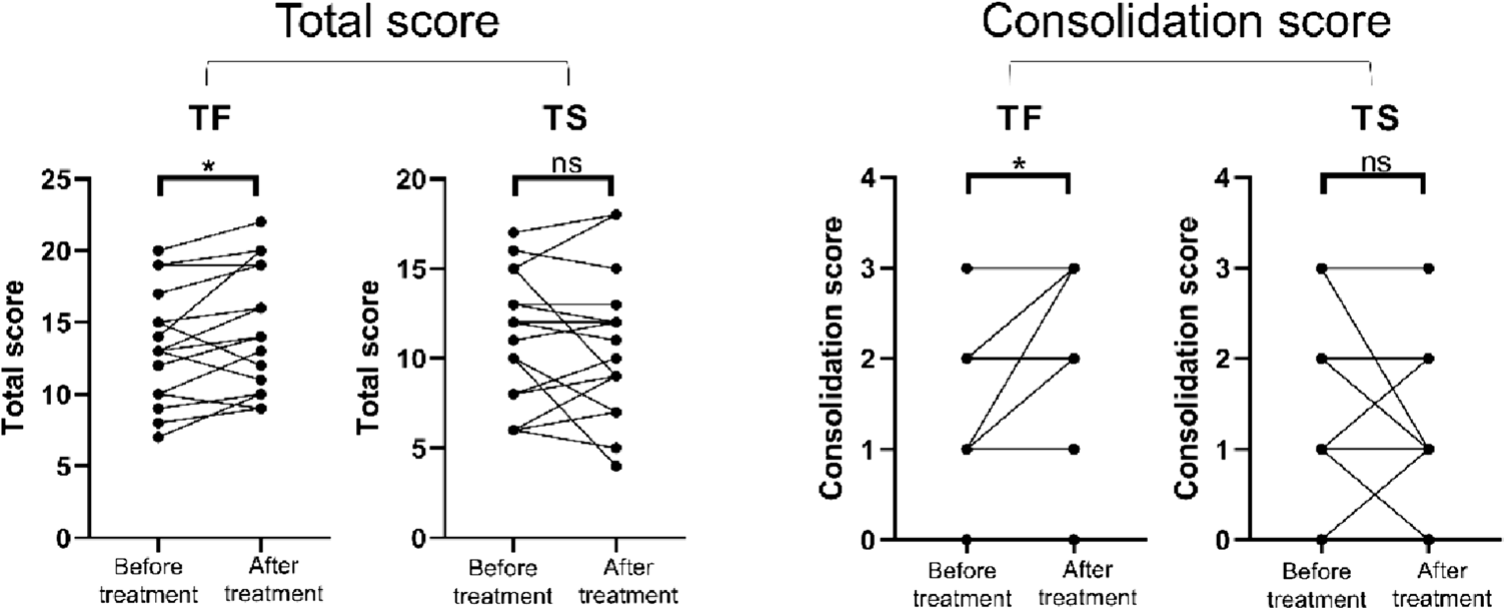
Scores changes between the two scans in total CT scores and consolidation score before and after treatment for *Mycobacterium abscessus* complex lung disease. Patients were classified into the treatment failure group (TF) or treatment success group (TS) based on the treatment outcome. The comparisons were analyzed using Wilcoxon test. *means *p* values < 0.05 and > 0.01. ns, non-significant

Comparing the interval changes of the initial and follow-up CT scores between the TF and TS groups, the changes of CT scores for consolidation (Figs. 3 and 4) and total score were significantly increased in patients in TF group but decrease in TS group [0 (0–1) vs. 0 (− 1–1), *p* = 0.024; 1 (0.5–2.5 vs. 0 (− 1–1), *p* = 0.049, respectively] (Table 4 and Fig. 5).

### Logistic regression analysis of predictors of treatment outcome

In univariate logistic regression for predictors of treatment failure, the pulmonary cavitation diameter (odds ratio [OR], 3.668; 95% CI 0.951–14.142 by 1-score increment; *p* = 0.05) and cavitation extension (OR, 2.512; 95% CI 1.028–6.139; *p* = 0.04 by 1-score increment) in the initial CT scan. In follow-up CT scan, cavitation diameter (OR, 2.83; 95% CI 1.058–7.570 by 1-score increment; *p* = 0.04), cavitation extension (OR, 2.357; 95% CI 1.066–5.211 by 1-score increment; *p* = 0.03), consolidation extension (OR, 2.495; 95% CI 1.134–5.490 by 1-score increment; *p* = 0.023), and total score (OR, 1.266; 95% CI 1.037–1.545 by 1-score increment; *p* = 0.021). The change in the consolidation extension score between initial and follow-up CT (OR, 9.094; CI 1.179–70.115 by 1-score increment; *p* = 0.04) were significant.

Multivariable logistic analysis was performed using the significant variables in univariate analysis. Cavity extension at initial CT (adjusted OR [aOR], 2.512; 95% CI 1.028–6.139 per 1-score increase; *p* = 0.04), consolidation extension at follow-up CT scan [(aOR), 2.495; 95% CI 1.134–5.490 per 1-score increase; *p* = 0.02], and the change in consolidation extension between initial and follow-up CT [(aOR), 9.094; CI 1.179–70.115 per 1-score increase; *p* = 0.04] were independent predictors of treatment failure.

### Receiver operating characteristic (ROC) curves for the predictors

**Fig. 6:**
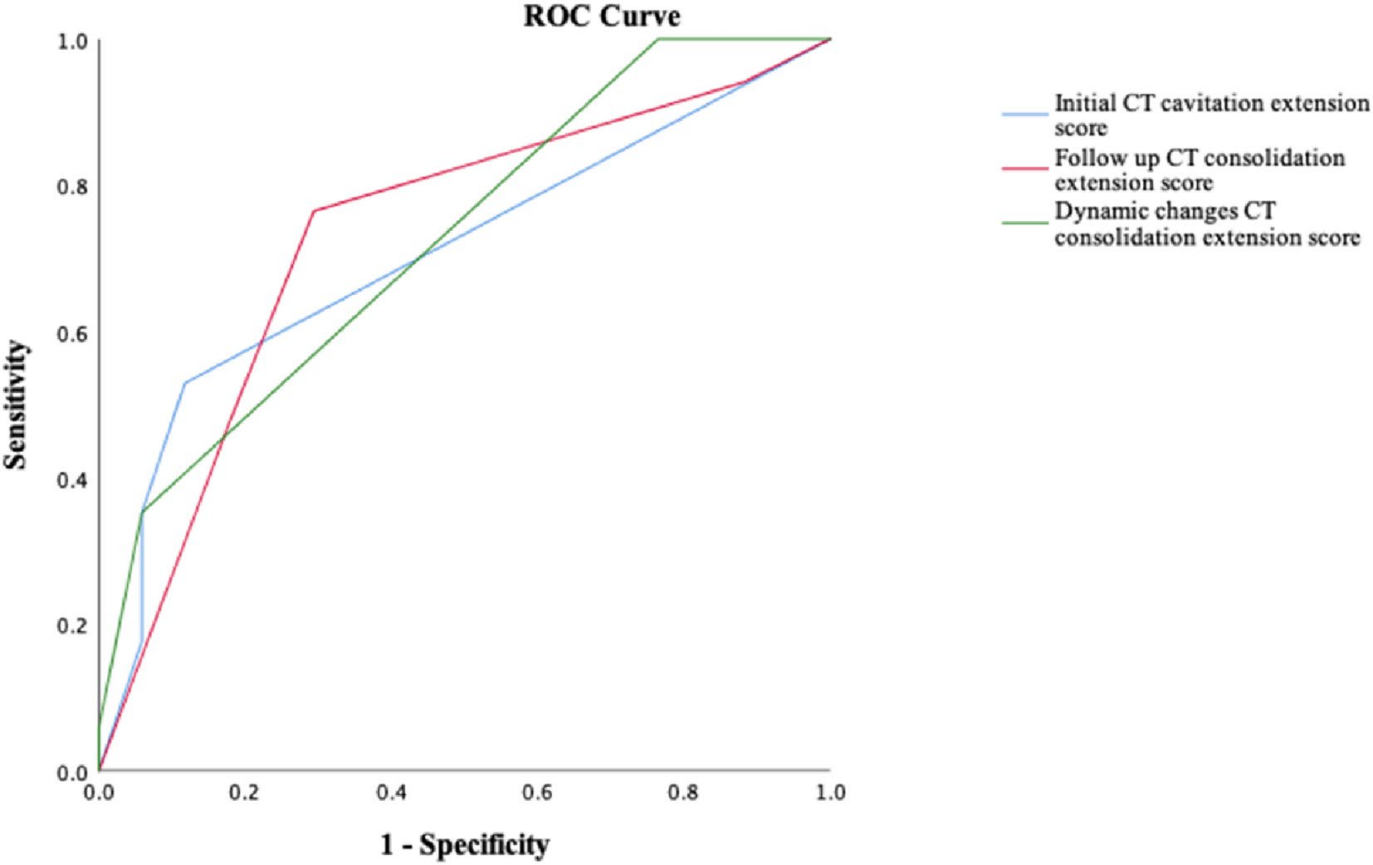
Receiver operating characteristic curves (ROC) for prediction of treatment failure outcome in *Mycobacterium abscessus* complex lung disease (MABC-LD). The areas under the curve for the cavitation extension score at the initial CT scan, the consolidation extension score at the follow-up CT scan, and the change in the interval consolidation extension score were 0.706 (95%CI 0.526–0.886), 0.730 (0.554–0.906), and 0.725 (0.556–0.894), respectively

The area under the curve (AUC) of the ROC curves for treatment failure was 0.706 (95% CI 0.526–0.886; sensitivity 52% and specificity 88% if score = 0.5) using cavitation extension at initial CT. The AUC of the ROC curves were 0.730 (95% CI 0.554–0.906; sensitivity 76.5% and specificity 70.6% if score = 1.5) for consolidation extension at follow-up CT, and 0.725 (95% CI 0.556–0.894; sensitivity 35.4%, specificity 94.1% if score = 0.5) for the change in consolidation extension between initial and follow-up CT (Fig. 6).

## Discussion

The present study observed a high treatment failure rate of 50% in MABC-LD. We semi-quantitatively assessed lung CT scores before and after treatment to identify predictors of treatment outcome in MABC-LD. High CT scores for cavitation extension at initiation of treatment, high scores for consolidation extension in follow-up, and an increase in the consolidation extension score during follow-up were independently associated the treatment failure outcome, with areas under the ROC of 0.706, 0.730, and 0.725, respectively.

Pulmonary cavitation is previously recognized as a predictor of a poor prognosis in NTM-LD [18]. Several studies indicate progressive cavitation is associated with the destruction of lung tissue, decreased lung function, respiratory failure, and a higher mortality rate compared to patients without cavities [19–21]. Higher virulence was observed in NTM-LD with cavities [13], in addition, unfavorable microbiological responses have also been reported in the fibrocavitary form of NTM-LD [21]. Although cavitation is less frequent in MABC-LD than MAC-LD [12], cavitation is still associated with poorer microbiological outcomes in MABC-LD, which is already difficult to treat due to multidrug resistance [22, 23]. Thus, recognition of cavitation in the early stages of treatment is important. Of the twelve patients (35.3%) with cavitation in the present study, nine (75%) had an unfavorable microbiological outcome. The AUC of the cavitation extension score was 0.706 with high specificity of 88% using a cutoff value of 0.5. Thus, observation of cavitation in patients with MABC-LD at the initial CT is a red-flag for treatment failure and suggests adequate standard treatment is warranted as early as possible.

The radiographic type of classification cannot accurately describe all of the complex patterns of NTM-LD. For example, although the fibrocavitary form of NTM-LD was associated with unfavorable microbiological responses [11, 21], cavities in the nodular bronchiectasis form of NTM-LD were also associated with a poorer prognosis compared to patients with NTM-LD without cavities [16]. Although the guidelines recommended prompt antibiotic treatment for the fibrocavitary form of NTM-LD, this treatment is also suggested for patients with cavities, even the nodular bronchiectatic form of NTM-LD [20]. Therefore, compared with the pattern approach of the type classification, our semi-quantitative CT scoring system provides a useful radiographic parameter for objective assessment of the severity and extension of the disease and may help to tailor the treatment.

Air-space consolidation in NTM-LD was first described in 1993 by Moore et al., who reported 29/40 cases (73%) had air-space consolidation [24]. Several reports have discussed the association between consolidation in NTM-LD and prognosis. Ushiki et al. [25] and Lee et al. [20] reported that cavitary and consolidated lesions are risk factors for progression in NTM-LD. Consolidation was a predictor of progressive cavities and associated with high mortality in MAC-LD [26]. Furthermore, the presence of consolidation at the end of treatment was suggested to be an independent predictive factor for recurrence in NTM-LD [27]. Similarly, our study found that a consolidation extension score ≥ 2 (sensitivity 76.5% and specificity 70.6%) or an increase in this score during treatment (specificity 94.1%) may be worrisome signs of treatment failure in MABC-LD.

This study has several limitations. First, the case number was small due to stringent inclusion criteria. However, the cases included for analysis might demonstrate a considerable level of homogeneity. This study still has its importance as MABC-LD is relatively difficult to treat, and monitoring during treatment may help to identify patients at risk of probable treatment failure. Our semi-quantitative CT scoring method can objectively identify high-risk patients at the start of treatment and during treatment. Second, the treatment regimens, data collection, and the timing of the chest CT performed were not standardized due to the retrospective nature of this study. The treatment duration may be prolonged due to the nature of treatment failure. Third, the subspecies of MABC, which have varying responses to macrolide treatment, were not fully available (only 32 cases were obtained) in this retrospective study. Furthermore, with only four cases identified as *Mycobacterium massiliense*, the differences in CT manifestations among subspecies could not be thoroughly assessed in this study. Fourth, this study was conducted in only two institutions in Taiwan. Thus, prospective studies are needed to validate our results in other populations and ethnicities.

In conclusion, the semi-quantitative CT scoring method proposed in this study might serve as an objective and faster tool to assist clinical physicians in objectively and swiftly evaluating the current condition of patients and adjusting treatment strategies in MABC-LD, a disease with high treatment failure rate (50%). Clinicians should consider high pre-treatment cavitation extension, high post-treatment consolidation scores, and increasing during-treatment consolidation score as alarm signs of treatment failure, because these factors are independently associated with unfavorable treatment outcomes.

### Supplementary Information

Below is the link to the electronic supplementary material.

Supplementary file 1 (DOCX 14 kb)

## Abbreviations

MABC-LD: *Mycobacterium abscessus* complex lung disease
NTM: Non-tuberculous mycobacteria
NTM-LD: Non-tuberculous mycobacterial lung disease
CT: Computed tomography
