# Supplementary material for: CT findings as predictive factors for treatment failure in Mycobacterium abscessus complex lung disease: a retrospective cohort study
Source: Jpn J Radiol. 2024 May 6;42(8):852–61. doi: 10.1007/s11604-024-01570-y (PMC11286655; doi:10.1007/s11604-024-01570-y)
Supplement: Supplementary file 1 — (DOCX 14 kb) [file 11604_2024_1570_MOESM1_ESM.docx]

**Supplemental Materials**

**Table S1.** Interobserver agreement for CT pattern of parenchymal lung abnormality.

| Pattern | Time point A ICC | Time point A  95% CI | Time point B ICC | Time point B  95% CI | *p*-value |
| --- | --- | --- | --- | --- | --- |
| Bronchiectasis severity | 0.975 | 0.951, 0 .988 | 0.945 | 0.893, 0.972 | <0.001 |
| Bronchiectasis extension | 0.889 | 0.789, 0 .943 | 0.897 | 0.805, 0.947 | <0.001 |
| Bronchiolitis severity | 0.758 | 0.568, 0.871 | 1 | 1, 1 | <0.001 |
| Bronchiolitis extension | 0.978 | 0.957, 0 .989 | 0.954 | 0.910, 0.977 | <0.001 |
| Cavitation size | 0.942 | 0.887, 0 .971 | 0.939 | 0.881, 0.969 | <0.001 |
| Cavitation extension | 0.987 | 0.974, 0 .993 | 0.977 | 0.955, 0 .988 | <0.001 |
| Consolidation extension | 0.894 | 0.799, 0 .946 | 0.845 | 0.712, 0.920 | <0.001 |
| Nodule extension | 0.825 | 0.678, 0 .909 | 0.873 | 0.761, 0 .935 | <0.001 |
| ICC, intraclass correlation coefficient; CI, confidence interval; CT, computed tomography | | | | |  |
